# Advantages of Efficacy and Safety of Fixed-Dose Tafluprost/Timolol Combination Over Fixed-Dose Latanoprost/Timolol Combination

**DOI:** 10.1371/journal.pone.0158797

**Published:** 2016-07-06

**Authors:** Masahiro Fuwa, Kenji Ueda, Takahiro Akaishi, Naoko Yamashita, Tomoko Kirihara, Atsushi Shimazaki, Hidetoshi Mano, Kouichi Kawazu

**Affiliations:** Global Research and Development, Santen Pharmaceutical Co., Ltd., Ikoma-shi, Nara, Japan; Bascom Palmer Eye Institute, University of Miami School of Medicine;, UNITED STATES

## Abstract

**Purpose:**

To compare the safety and efficacy of fixed-dose tafluprost/timolol combination (Taf/T-FDC) with those of fixed-dose latanoprost/timolol combination (Lat/T-FDC) by measuring the intraocular pressure (IOP)-lowering effect, ocular pharmacokinetics, and ocular surface toxicity.

**Methods:**

The IOP-lowering effect of Taf/T-FDC and Lat/T-FDC in ocular normotensive monkeys was evaluated at 4 and 8 h after instillation in study A, at 12, 14, 16, and 18 h after instillation in study B, and at 24, 26, 28, and 30 h after instillation in study C. Drug penetration into the eye was evaluated by measuring the concentrations of timolol, tafluprost acid (active metabolic form of tafluprost), and latanoprost acid (active metabolic form of latanoprost) using liquid chromatography coupled with tandem mass spectrometry after single instillation of Taf/T-FDC or Lat/T-FDC to Sprague Dawley rats. Cytotoxicity following 1–30 min exposure of SV40-transformed human corneal epithelial cells to Taf/T-FDC or Lat/T-FDC was analyzed using 3-(4,5-dimethylthiazol-2-yl)-5-(3-carboxymethoxyphenyl)-2-(4-sulfophenyl)-2H-tetrazolium assays. Undiluted and 10-fold diluted solutions of each FDC were evaluated.

**Results:**

The IOP-lowering effect of Taf/T-FDC was almost equivalent to that of Lat/T-FDC at 4–8 h after instillation. The peak IOP reduction of Taf/T-FDC and Lat/T-FDC was observed at 8 h after instillation, and there is no difference between the two. The difference between them was observed at 24–30 h after instillation, and Taf/T-FDC demonstrated a significantly greater IOP-lowering effect than Lat/T-FDC at 24–30 h after instillation. The IOP-lowering effect of Taf/T-FDC was sustained up to 30 h after instillation, while that of Lat/T-FDC had almost disappeared at 28 h after instillation. Timolol concentrations in aqueous humor after Taf/T-FDC instillation were higher than those after Lat/T-FDC instillation (C_max_, 3870 ng/mL *vs* 1330 ng/mL; AUC_inf_, 3970 ng·h/mL *vs* 1250 ng·h/mL). The concentrations of tafluprost acid and latanoprost acid in aqueous humor after instillation of Taf/T-FDC and Lat/T-FDC, respectively, were similar to those after instillation of mono-preparations of tafluprost and latanoprost, respectively. The cytotoxic effect of Taf/T-FDC to the human corneal epithelial cells was significantly lower than that of Lat/T-FDC at all evaluated time points in both undiluted and 10-fold diluted FDCs.

**Conclusion:**

Taf/T-FDC provides increased IOP-lowering effect duration and lower potential ocular surface toxicity than Lat/T-FDC.

## Introduction

Glaucoma, a neurodegenerative ocular disease characterized by selective loss of retinal ganglion cells (RGCs) and resultant visual field defects, is the second leading cause of blindness worldwide. Elevated intraocular pressure (IOP) is a major risk factor for glaucoma progression [1, 2], and IOP reduction is the main target of glaucoma treatment [2, 3].

Glaucoma patients are initially treated with pharmacological monotherapy, such as prostaglandin analogue (PGA) or beta-adrenergic antagonist; however, in more than 40% patients, additional IOP-lowering drugs are required to control their IOP; hence, two or more drugs are used in the treatment [4–6]. However, the addition of a second drug increases the complexity of treatment regimens and incidence of dosing errors [7, 8]. As a result, separate instillation times (by at least 5–10 min) are recommended to avoid diluting and/or washing of either drug from cul-de-sac [9]; this approach is very inconvenient for patients. Therefore, there is a significant clinical concern regarding reduction in the pharmacological effects of instilled drugs in patients who do not follow these recommendations. Fixed-dose combination (FDC) therapy is a solution for improving patient compliance [10]. Francis *et al*. indicated that FDC therapies provide better IOP control than unfixed combinations therapies in real-life settings [11].

In recent years, several FDCs of routinely-used IOP-lowering drugs have been developed [12], such as beta-adrenergic antagonist or alpha-2-adrenergic agonist with carbonic anhydrase inhibitor and PGA with beta-adrenergic antagonist, with the aim of maximizing patient medication adherence. Among the recently developed FDCs, those comprising a PGA and beta-adrenergic antagonist (PGA/beta-FDCs) are attracting considerable attention because these therapies combine two different mechanism of action and are most commonly used to treat glaucoma, expecting strong IOP reduction [13]. Ocular surface disease (OSD) is a common complication of chronic treatment with preserved topical IOP-lowering drugs in patients with glaucoma or ocular hypertension. The reported prevalence of OSD in glaucoma patients varies between 45% and 60% [14–16]. FDCs decrease daily exposure to preservatives, such as benzalkonium chloride (BAK), which potentially have toxic effects on the ocular surface and increase treatment side effects [17]. Therefore, in addition to the medication adherence, FDCs are expected to reduce the possibility developing OSD.

An FDC of tafluprost and timolol (Taf/T-FDC) is a recently developed PGA/beta-FDC and is expected to become a new treatment choice for glaucoma. Taf/T-FDC demonstrated an almost equivalent IOP-lowering effect to concomitant instillation of 0.0015% tafluprost and 0.5% timolol in a recent clinical trial [18]. Latanoprost/timolol FDC (Lat/T-FDC), an FDC that combines 0.005% latanoprost and 0.5% timolol, was the first PGA/beta-FDC to be marketed worldwide; however, the IOP-lowering effect of Lat/T-FDC was considered lower than that of concomitant administration of timolol and latanoprost [19, 20]. Diestelhorst *et al*. reported that a mean IOP at baseline was 16.9 mmHg in both study groups (Lat/T-FDC group and latanoprost/timolol unfixed combination group) and the mean diurnal IOP was 17.0 mmHg after Lat/T-FDC treatment and 15.9 mmHg after latanoprost/timolol unfixed combination therapy (P < 0.0001) [20]. Therefore, there is a possibility that the efficacy of Taf/T-FDC and Lat/T-FDC is different; however, differences in the profile of efficacy and safety of these FDCs have yet to be fully elucidated.

In the present study, the IOP-lowering effect (strength and duration), ocular pharmacokinetics, and ocular surface toxicity of Taf/T-FDC and Lat/T-FDC were compared to clarify the characteristics of efficacy and safety of these two FDCs.

## Materials and Methods

### Drugs

TAPCOM^®^ combination ophthalmic solution (Taf/T-FDC; 0.0015% tafluprost and 0.5% timolol preserved with 0.001% BAK) and TAPROS^®^ ophthalmic solution 0.0015% (0.0015% tafluprost preserved with 0.001% BAK) were supplied by Santen Pharmaceutical Co., Ltd. (Osaka, Japan). Xalacom^®^ combination eye drops (Lat/T-FDC; 0.005% latanoprost, 0.5% timolol preserved with 0.02% BAK) and Xalatan^®^ eye drops 0.005% (0.005% latanoprost preserved with 0.02% BAK) were purchased from Pfizer Inc. (New York, NY, U.S.A.).

Timolol and tafluprost acid, used as standards in quantitative analyses, were provided by Santen Pharmaceutical Co., Ltd. (Osaka, Japan). Latanoprost acid was purchased from Sigma-Aldrich (St. Louis, MO, U.S.A.).

### Animals

Female Sprague Dawley rats (Charles River Laboratories Japan, Inc., Yokohama, Japan) weighing 155–214 g and male cynomolgus monkeys (Keari Co., Ltd., Osaka, Japan and Shin Nippon Biomedical Laboratories, Ltd, Tokyo, Japan) weighing 6.3–10.8 kg were used in the present study. Animals were housed under a 12-h light–dark cycle. All monkeys were housed individually, were provided 100 g of Monkey Bit (Nippon Nosan, Kanagawa, Japan) per day and water ad libitum, and received environmental enrichment including toys such as mirrors. They were monitored once daily by breeding stuff belonging to the Animal Care Team at Santen Pharmaceutical Co., Ltd. Breeding environment was set as follows: temperature and humidity were controlled at 23°C (acceptable range: 18–28°C) and 50% (acceptable range: 30%–70%), respectively. The monkeys used for IOP measurement were included following acclimations to prevent stress from measuring IOP; following was the acclimatization protocol: 1 week acclimation for sitting Monkey chair and 3–5 months tonometry acclimation. Local anesthesia was administered prior to measuring IOP. After the end of the study, all monkeys were continuously kept for use in other experiments. For sacrifice, the rats were euthanized by inhalation of isoflurane anesthesia (ISOFLURANE inhalation solution; Pfizer Inc., New York, NY, U.S.A.) and were exsanguinated from the abdominal aorta. No animals became ill, showed abnormalities in health conditions, or died prior to the experimental endpoint, therefore there were no animals that received medical treatment or humane euthanasia.

All animal care and experimental procedures were performed in accordance with the ARVO Statement for the Use of Animals in Ophthalmic and Vision Research and were approved and monitored by the Animal Care and Use Committee at Santen Pharmaceutical Co., Ltd (approval number: DR-2013-0007, DR-2013-0413, DR-2014-0044, DR-2014-0180, and DR-2015-0023).

### IOP measurement

Ocular normotensive male cynomolgus monkeys were used in all studies. Before the study, all monkeys were trained in being restrained in a monkey chair (CL-4535; Primate Products, Miami, FL, U.S.A.) and undergoing IOP measurements without any general anesthesia or sedation. For IOP measurements, each monkey was kept in a sitting position in the monkey chair and IOP was measured with a pneumatonograph (Model 30 Classic^TM^ Pneumatonometer; Reichert Technologies, NY, U.S.A.). For local corneal anesthesia, 0.4% oxybuprocaine solution (Benoxil^®^ ophthalmic solution 0.4%; Santen Pharmaceutical Co., Ltd., Osaka, Japan) was topically applied before IOP measurements. One drop of oxybuprocaine was administered per eye per animal. We administered it twice before IOP measurement (3–20 min before and just before the measurement). All IOP measurement studies were performed under blind conditions.

### IOP-lowering effects of Taf/T-FDC and Lat/T-FDC

**Fig 1:**
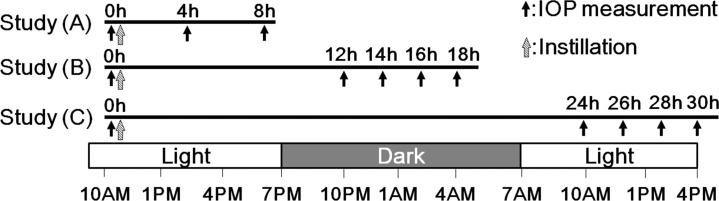
Timeline of the three studies of Taf/T-FDC and Lat/T-FDC in ocular normotensive monkeys. Three independent IOP studies were performed for the comparison of the IOP-lowering effects of Taf/T-FDC and Lat/T-FDC in ocular normotensive monkeys. In all studies, drug instillations were performed at 10:00 AM to 11:00 AM. IOP measurements were performed immediately before, and at 4 and 8 h after drug instillation in study A, immediately before, and at 12, 14, 16, and 18 h after drug instillation in study B, and immediately before, and at 24, 26, 28, and 30 h after drug instillation in study C. Dark-phase IOP measurements were performed under a safelight.

Three independent studies were performed according to the schedules described in Fig 1. In all studies, drugs or saline were instilled to the right eye of each monkey between 10:00 AM and 11:00 AM, with the contralateral eye remaining untreated. IOP measurements were performed immediately before, and at 4 and 8 h after, drug instillation in study A; immediately before, and at 12, 14, 16, and 18 h after, drug instillation in study B; and immediately before, and at 24, 26, 28, and 30 h after, drug instillation in study C. Dark-phase IOP measurements were performed under a safelight so as to not influence the circadian rhythm.

For studies A and B, 18 monkeys were used with 6 monkeys assigned to each of three groups in such a way that IOP values immediately before the initiation of the study were approximately equal among the three groups. In regard to the use of 9 animals in study C, the monkeys were firstly divided into 3 groups based on IOP values at baseline. Saline, Taf/T-FDC, and Lat/T-FDC were administered to each group, and IOP was measured at 24, 26, 28, and 30 h after treatment. Monkeys received each treatment in differing orders such that all monkeys received saline, Taf/T-FDC, and Lat/T-FDC. The mean changes in IOP were expressed as the means ± standard error of the mean (SEM).

### Ocular penetration after ocular instillation of ophthalmic solutions

Thirty-six rats were used for the timolol ocular penetration study of Taf/T-FDC and Lat/T-FDC. In each group, aqueous humor was collected at 5, 15, and 30 min, and 1, 2, and 4 h after instillation (n = 6 eyes per time point). Sixty rats were used for the ocular penetration study of FDCs and mono-preparations of tafluprost and latanoprost. In each group, aqueous humor was collected at 5, 15, and 30 min, and 1 and 2 h after instillation (n = 6 eyes per time point).

Timolol, tafluprost acid (active metabolic form of tafluprost), and latanoprost acid (active metabolic form of latanoprost) in the collected aqueous humor were quantified by liquid chromatography (Nexera; Shimadzu Corporation, Kyoto, Japan) coupled with tandem mass spectrometry (QTRAP5500; AB Sciex, Framingham, MA, U.S.A.). All quantitative analyses were conducted using validated methods.

Maximum concentrations of timolol, tafluprost acid, and latanoprost acid in the aqueous humor (C_max_) were calculated from mean concentrations. T_max_ was defined as the time to reach C_max_. Areas under time-aqueous humor concentration curves up to 2–4 h after instillation (AUC_0–2h_ or AUC_0–4h_) were calculated from mean concentrations using the linear trapezoidal method. Elimination half-life (T_1/2_) values were obtained by dividing the logarithm of 2 by the terminal elimination rate constant. AUC values were extrapolated to infinity by dividing the last measurable concentration by the terminal elimination rate constant. All parameters were determined using non-compartmental pharmacokinetic analyses (Phoenix WinNonlin Version 6.3; Certara LP, Princeton, NJ, U.S.A.).

### In vitro cytotoxicity study

#### Testing solutions

Taf/T-FDC and Lat/T-FDC were tested at both undiluted and 10-fold diluted concentrations in low-glucose Dulbecco’s modified Eagle medium and Ham’s Nutrient mixture F-12 (DMEM/F12; Life Technologies, NY, U.S.A.).

#### Culture of HCE-T cells

SV40-transformed human corneal epithelial (HCE-T; RIKEN BRC, Tsukuba, Japan) cells were cultured in DMEM/F12 supplemented with 15% fetal bovine serum (FBS), 5-μg/mL insulin (Akron Biotechnology, LLC, FL, U.S.A.), 40-μg/mL gentamicin (Invitrogen, CA, U.S.A.), and 10-ng/mL epidermal growth factor (EGF; BD Biosciences, CA, U.S.A.) in an incubator (5% CO_2_, 37°C) [21].

#### MTS assay procedures

Cell viability was measured using 3-(4,5-dimethylthiazol-2-yl)-5-(3-carboxymethoxyphenyl)-2-(4-sulfophenyl)-2H-tetrazolium (MTS) assays (CellTiter 96 Aqueous One Solution Cell Proliferation Assay, Promega, WI, U.S.A.) according to the manufacturer’s instructions. Briefly, HCE-T cells were seeded in 96-well culture plates (1 x 10^4^ cells/well) and incubated in DMEM/F12 supplemented with 10% FBS for 72 h. Subsequently, culture medium was removed and cells were washed once with DMEM/F12 before the addition of 100 μL of test solution (undiluted or 10-fold diluted solutions). MTS assays were performed at three incubation time points (1, 3, and 5 min) for undiluted test solutions and four incubation time points (5, 10, 15, and 30 min) for 10-fold diluted test solutions. Test solutions were removed and 20 μL of MTS reagent in addition to 100 μL of DMEM/F12 were added to each well. HCE-T cells were incubated at 37°C for 2 h. Subsequently, absorbance values at 490 nm were recorded using a spectrometer. MTS assay procedures were performed in 8 replicates per each group. Cell viability values were expressed as percentages of mean control (DMEM/F12) values in each measurement point and as means ± standard deviation (SD).

### Statistical analysis

All statistical analyses were performed using EXSUS software version 8.0.0 (CAC EXICARE Corporation, Tokyo, Japan). Tukey’s multiple comparison test was used for comparisons among all groups in IOP study and in vitro cytotoxicity study.

## Results

### IOP lowering effect of Taf/T-FDC versus Lat/T-FDC

**Fig 2:**
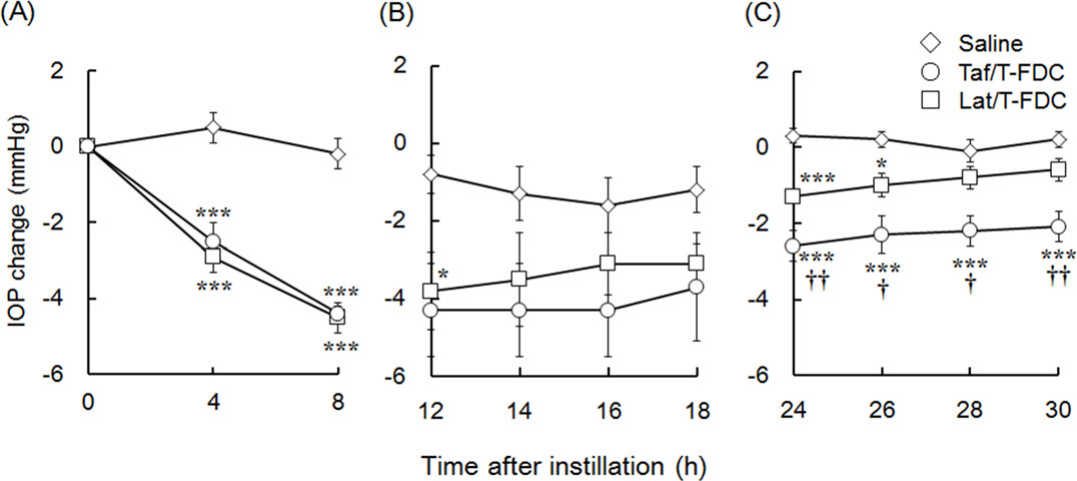
Effects of topical instillation of Taf/T-FDC and Lat/T-FDC on intraocular pressure (IOP) in ocular normotensive monkeys. Saline (20 μL), Taf/T-FDC (20 μL), or Lat/T-FDC (20 μL) were topically instilled to right eyes, while left eyes remained untreated. IOP was measured using a pneumatic tonometer. Changes in IOP were calculated as the difference from pre-instillation IOP values at the 0 h time point. (A, B) Data represent the mean IOP change ± SEM of 5–6 eyes (saline-treated group and Taf/T-FDC-treated group, n = 6; Lat/T-FDC treated group, n = 5). (C) Data represent the mean change in IOP ± SEM of 9 eyes. ^*^ P < 0.05, ^***^ P < 0.001 vs Saline, ^†^P < 0.05, ^††^P < 0.01 vs Lat/T-FDC according to Tukey’s multiple comparison test.

Three independent studies were performed based on the schedules shown in Fig 1 in order to compare the IOP-lowering effects of Taf/T-FDC and Lat/T-FDC over early (0–8 h), middle (12–18 h), and late (24–30 h) terms. Changes in IOP are shown in Fig 2 (see data in S1 File). No statistically significant differences in initial IOP values were observed in any of three studies. The mean initial IOP values of each group were 16.7 mmHg to 17.6 mmHg in study A (n = 5–6), 17.9 mmHg to 18.1 mmHg in study B (n = 5–6), and 17.6 mmHg to 17.8 mmHg in study C (n = 9). During this study, no significant abnormalities in ocular condition or general health were observed. In study A and B, one monkey in the Lat/T-FDC group was excluded as accurate measurements could not be obtained from this animal.

Taf/T-FDC demonstrated an IOP-lowering effect until 30 h after instillation, with significant differences observed at 4, 8, 24, 26, 28, and 30 h after instillation. Lat/T-FDC demonstrated an IOP-lowering effect until 26 h after instillation, with significant differences observed at 4, 8, 12, 24, and 26 h after instillation. The peak IOP-lowering effect was detected at 8 h after instillation in both Taf/T-FDC and Lat/T-FDC groups, with no statistically significant difference (P > 0.05) observed between Taf/T-FDC and Lat/T-FDC. The mean changes in IOP at this time (8 h after instillation) in the Taf/T-FDC and Lat/T-FDC groups were −4.4 ± 0.2 mmHg (25% reduction from initial IOP values) and −4.5 ± 0.4 mmHg (26% reduction from initial IOP values), respectively (Fig 2A and S1 File). After appearance of the peak IOP-lowering effect, Taf/T-FDC group continued lowering IOP until 16 h after instillation. On the other hand, Lat/T-FDC group started to return at 12 h after instillation (Fig 2B and S1 File).

When comparing the duration of the IOP-lowering effect between the Taf/T-FDC and Lat/T-FDC groups, Taf/T-FDC demonstrated a significantly greater IOP-lowering effect than Lat/T-FDC at 24 h after instillation and onwards. Changes in IOP in the Taf/T-FDC and Lat/T-FDC groups were −2.6 ± 0.4 mmHg and −1.3 ± 0.2 mmHg at 24 h after instillation, −2.3 ± 0.5 mmHg and −1.0 ± 0.3 mmHg at 26 h after instillation, −2.2 ± 0.4 mmHg and −0.8 ± 0.3 mmHg at 28 h after instillation, and −2.1 ± 0.4 mmHg and −0.6 ± 0.3 mmHg at 30 h after instillation, respectively (Fig 2C and S1 File). The IOP-lowering effect of Lat/T-FDC had almost disappeared at 28 h after instillation; however, the IOP-lowering effect of Taf/T-FDC remained at 30 h after instillation.

The results of three studies demonstrated Taf/T-FDC had greater efficacy than Lat/T-FDC in terms of the duration of the IOP-lowering effect.

### Drug penetration to anterior chamber after ocular instillation of Taf/T-FDC and Lat/T-FDC

**Fig 3:**
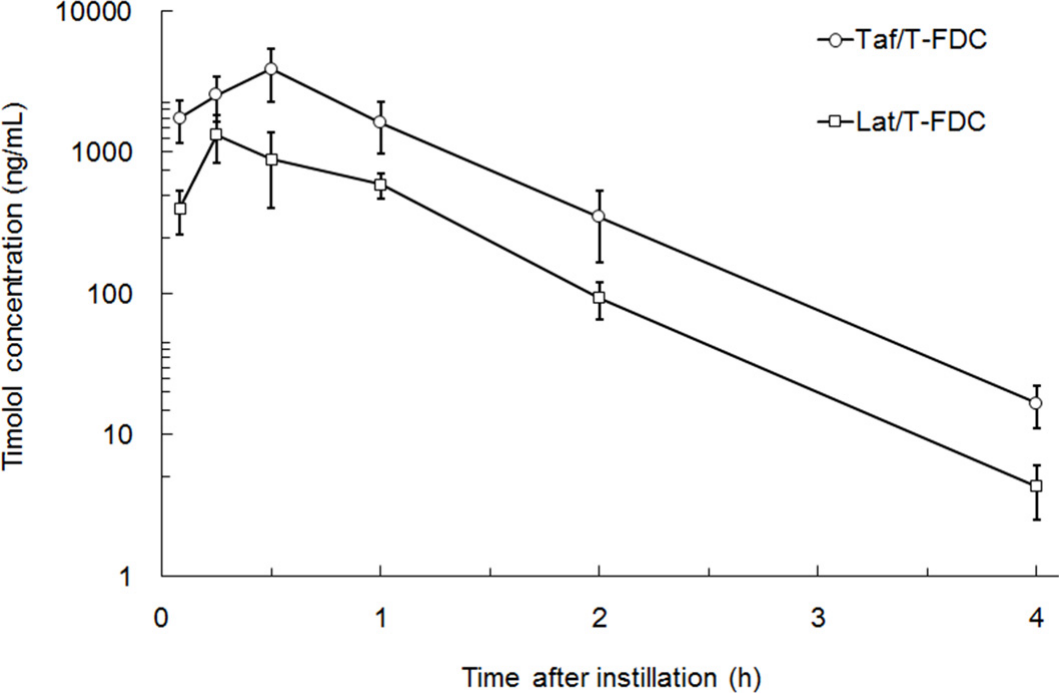
Timolol concentrations in aqueous humor after instillation of Taf/T-FDC and Lat/T-FDC (mean ± SD, n = 6 eyes).

Ocular penetration of timolol was compared between Taf/T-FDC and Lat/T-FDC (Fig 3 and S2 File). After instillation of Taf/T-FDC, timolol concentrations reached C_max_ at 0.5 h and declined with a T_1/2_ of 0.454 h. Concentrations were consistently higher after instillation of Taf/T-FDC than after instillation of Lat/T-FDC. The C_max_ and AUC_inf_ of timolol after instillation of Taf/T-FDC were 2.9 times and 3.2 times higher than after instillation of Lat/T-FDC, respectively (Table 1).

**Table 1:** Pharmacokinetic parameters of timolol in aqueous humor after instillation of Taf/T-FDC and Lat/T-FDC.

Ophthalmic solution	C_max_(ng/g)	T_max_(h)	AUC_0-4h_(ng·h/mL)	AUC_inf_(ng·h/mL)	T_1/2_(h)
Taf/T-FDC	3870	0.5	3960	3970	0.454
Lat/T-FDC	1330	0.25	1250	1250	0.426

Each parameter was calculated using mean concentrations of timolol (n = 6 eyes).

**Fig 4:**
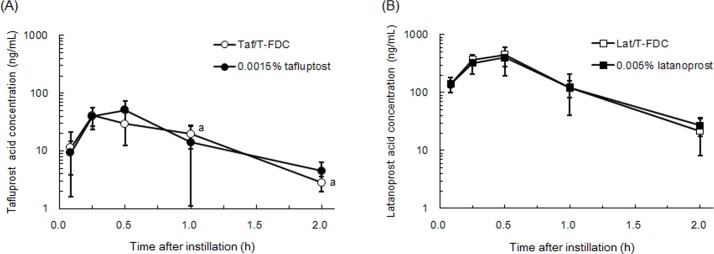
Comparison of aqueous humor concentrations of acid forms of PGAs after instillation of FDCs and mono-preparations (mean ± SD, n = 6; ^a^n = 5 eyes). (A) Tafluprost acid concentration profiles after instillation of Taf/T-FDC and 0.0015% tafluprost ophthalmic solution. (B) Latanoprost acid concentrations after instillation of Lat/T-FDC and 0.005% latanoprost ophthalmic solution.

In addition, the ocular penetration of PGAs was compared between FDCs and mono-preparations. After instillation of FDCs and mono-preparations, the aqueous humor concentrations of acid forms, the pharmacologically active forms of PGA generated by hydrolysis in the cornea, were measured and compared (Fig 4 and S3 File). The pharmacokinetic parameters of acid forms are shown in Table 2. Tafluprost acid concentrations demonstrated similar profiles after instillation of Taf/T-FDC and tafluprost mono-preparation, reaching C_max_ at 0.25–0.5 h and declining with a T_1/2_ of 0.43–0.45 h. Latanoprost acid concentrations, which also demonstrated similar profiles after instillation of Lat/T-FDC and latanoprost mono-preparation, reached C_max_ at 0.5 h and declined with a T_1/2_ of 0.35–0.40 h.

**Table 2:** Pharmacokinetic parameters of tafluprost acid and latanoprost acid in aqueous humor after instillation of FDCs and mono-preparations.

Ophthalmic solution	Analyte	T_max_(h)	C_max_(ng/mL)	AUC_0-2h_(ng∙h/mL)	AUC_inf_(ng∙h/mL)	T_1/2_(h)
Taf/T-FDC	Tafluprost acid	0.25	41.2	37.1	38.8	0.427
0.0015% tafluprost	Tafluprost acid	0.5	51.0	41.4	44.3	0.448
Lat/T-FDC	Latanoprost acid	0.5	449	364	374	0.350
0.005% latanoprost	Latanoprost acid	0.5	401	341	357	0.395

Each parameter was calculated using mean concentrations of Tafluprost acid or Latanoprost acid (n = 6 eyes).

### Comparison of the cytotoxicity of Taf/T-FDC and Lat/T-FDC on HCE-T cells

**Fig 5:**
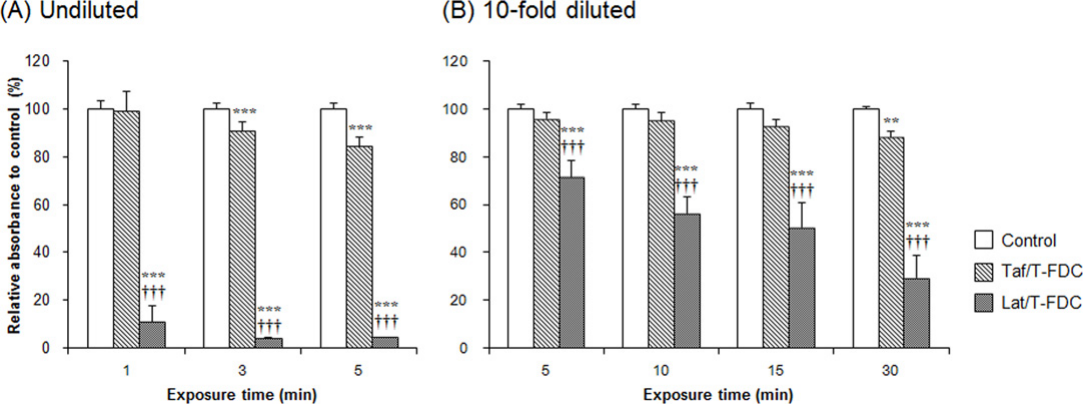
Cytotoxicity of Taf/T-FDC and Lat/T-FDC on HCE-T cells. MTS assays were performed at three incubation time points (1, 3, and 5 min) for undiluted Taf/T-FDC and Lat/T-FDC (A) and four incubation time points (5, 10, 15, and 30 min) for 10-fold diluted Taf/T-FDC and Lat/T-FDC (B), respectively. Medium (DMEM/F12) alone was used as a control. Cell viability was expressed as percent of control. Data represent the mean ± SD of 8 replicates. ^**^ P < 0.01, ^***^ P < 0.001 vs control, ^†††^P < 0.001 vs Taf/T-FDC according to Tukey’s multiple comparison test.

After 1, 3 and, 5 min exposure to undiluted drugs, Lat/T-FDC demonstrated a significant decrease in cell viability at all evaluated time points compared to the control group: 10.7 ± 6.8% at 1 min; 4.0 ± 0.2% at 3 min; and 4.3 ± 0.3% at 5 min exposure, respectively. On the other hand, Taf/T-FDC demonstrated no significant decrease at 1 min exposure (99.2 ± 8.2%), and significant decreases at 3 and 5 min exposure (91.0 ± 3.6% and 84.2 ± 4.0%, respectively) however, these decreases were significantly smaller than with Lat/T-FDC (Fig 5A and S4 File).

After 5, 10, 15, and 30 min exposure to 10-fold diluted drugs, Lat/T-FDC demonstrated significant decreases in cell viability at all evaluated time points compared to the control group: 71.6 ± 6.9% at 5 min; 56.5 ± 6.7% at 10 min; 50.3 ± 10.3% at 15 min; and 29.4 ± 9.2% at 30 min exposure, respectively. Taf/T-FDC demonstrated no significant decrease until 15 min exposure (95.9 ± 2.9% at 5 min; 95.1 ± 3.3% at 10 min; and 92.8 ± 3.0% at 15 min, respectively), with a significant decrease observed at 30 min exposure only (88.5 ± 2.1%), however, these decreases were significantly smaller than with Lat/T-FDC (Fig 5B and S4 File).

Taf/T-FDC demonstrated significantly lower cytotoxicity than Lat/T-FDC at all evaluated time points and two concentrations.

## Discussion

The strength and durability of the IOP-lowering effects of Lat/T-FDC and Taf/T-FDC were examined in the present study (Fig 2 and S1 File). In those experiments, Taf/T-FDC demonstrated greater IOP-lowering effect duration that was maintained until 30 h than Lat/T-FDC. These data demonstrated the superiority of Taf/T-FDC over Lat/T-FDC in terms of efficacy duration. Two factors that affect the duration of IOP-lowering effects were considered in the present study: the efficacy of each mono-preparation, tafluprost and latanoprost, and pharmacokinetics based on drug formulation. From the view point of the efficacy of each mono-preparation, it is thought that the difference in efficacy duration is attributable to the difference between the tafluprost and latanoprost compositions of Taf/T-FDC and Lat/T-FDC, respectively. A previous study [22] showed no significant difference between tafluprost and latanoprost in IOP reduction 24 h after a single instillation in monkeys. In the present study, concentrations of PGA acid forms in aqueous humor were measured after instillation of mono-preparations and FDCs containing tafluprost or latanoprost to examine the possibility that the difference of ocular penetration of PGAs between mono-preparation and FDCs caused the superior efficacy duration of Taf/T-FDC. The result showed there were no differences of ocular penetrations of PGAs between mono-preparation and FDCs (Fig 4 and S3 File). Therefore, it is considered that the difference in efficacy duration between Taf/T-FDC and Lat/T-FDC is unlikely to be attributable to the efficacy of tafluprost and latanoprost in FDCs.

From the view point of the pharmacokinetics based on drug formulation, Taf/T-FDC formulation was designed to maintain IOP-lowering effect when timolol instillation is changed from twice to once daily with FDC, on the bases that timolol ocular penetration increases with increased pH [23, 24], and the stability of tafluprost decreases with increased pH. As a result, timolol penetration into the eye with Taf/T-FDC has been reported higher than that with timolol mono-preparation and close to that with timolol gel-forming formulation [24]. In the present study, we measured timolol concentrations in aqueous humor after instillation of FDCs to examine the difference between Taf/T-FDC and Lat/T-FDC in timolol ocular penetration. As a result, the timolol ocular penetration was found to be greater for Taf/T-FDC than Lat/T-FDC (Fig 3 and S2 File), and it is probably due to difference in pH between Taf/T-FDC and Lat/T-FDC (6.7–7.2 and 5.8–6.2, respectively). These findings suggest that superior timolol penetration into the eye with Taf/T-FDC was possibly attribute to the long duration of the IOP-lowering effects with Taf/T-FDC.

In addition to pH, there are some formulations (e.g., gellan gum and sorbate formulation) that increase timolol concentration in aqueous humor. Higashiyama *et al*. showed that the ocular penetration of timolol was improved by the presence of sorbic acid in an ophthalmic solution and concluded that the improvement was caused by increasing the timolol distribution to corneal epithelium owing to the formation of more lipophilic ion pair complexes with sorbic acid [25]. Rozier *et al*. reported that the ocular penetration of timolol in an ophthalmic solution containing gellan gum was improved by the prolongation of precorneal residence time caused by gel formation in the conjunctival sac [26]. Once-daily timolol ophthalmic solutions containing gellan gum (Timoptic-XE®) or sorbate (Istalol®) has been reported to be at least as effective as timolol maleate solution given twice daily [27–29]. These reports indicate the similar efficacy of once-daily and twice-daily formulations is attributable to higher timolol concentrations in the aqueous humor following instillation of once-daily formulation. Therefore, the greater duration of the IOP-lowering effect of Taf/T-FDC compared to Lat/T-FDC is likely to be due to a higher timolol penetration with Taf/T-FDC.

In fact, Taf/T-FDC was shown to have a comparable IOP-lowering effect to concomitant instillation of 0.5% timolol and 0.0015% tafluprost in a recent clinical trial [18]. On the other hand, Lat/T-FDC reportedly has a slightly weaker effect than concomitant instillation of timolol and latanoprost [19, 20]. Consequently, Taf/T-FDC may be superior to Lat/T-FDC in terms of efficacy duration due to formulation improvement of appropriate pH setting, leading to improved efficacy.

Single instillation of Taf/T-FDC was found to lower IOP for greater than 30 h. IOP reduction is less affected by neglect of instillation for IOP-lowering drugs with greater efficacy duration. The prolonged duration of action indicates that an occasional missed dose may not induce substantial IOP variations. Such IOP fluctuations have been identified as a risk factor for glaucoma progression [30]. Therefore, the longer duration of IOP reduction by Taf/T-FDC may contribute to stable IOP control in patients and reduce the progression of glaucoma. The results of the present study demonstrated the superiority of Taf/T-FDC compared to Lat/T-FDC, corroborating the findings of previous clinical trials [18, 20, 31, 32].

We also compared the effect of the two FDCs, Taf/T-FDC and Lat/T-FDC, on the health of the ocular surface using *in vitro* cytotoxicity assays. Chronic use of topical anti-glaucoma drugs induces side effects, such as ocular inflammation, allergy, dry eye syndrome, and failure of filtration surgery [33–35], which can be caused by medication components such as additives, and there is a clinical need for IOP-lowering drugs without corneal toxicity or significantly reduced such effects. In the present study, Taf/T-FDC demonstrated significantly lower cytotoxicity than Lat/T-FDC when both undiluted drugs and 10-fold dilutions were used (Fig 5 and S4 File). Nelson reported the tear turnover rate of 0–5 minutes and 5–20 minutes estimated by the instillation of disodium fluorescein (DSF) and carboxyfluorescein (CF) [36]. We have calculated the area under the tear concentration-time curve (AUC) using the tear turnover rate reported by Nelson and tear volume [36]. When eye drops at a concentration of 100 are instilled, AUC_inf_ are estimated to be 215 and 141 using tear turnover rate of DSF and CF, respectively. These values are equal to the AUC with 22-minute and 14-minute exposure, respectively, of 10-fold-diluted eye drops. Therefore, we set the assay concentration and exposure time for MTS assay. When 10-fold dilutions were used, Lat/T-FDC demonstrated a significantly higher cytotoxicity than Taf/T-FDC. We considered two factors that may affect the cytotoxicity; the toxicity of the active pharmaceutical ingredient (API) and the cytotoxicity of additives. In the previous study, Pauly *et al*. reported that cytotoxicity was caused not by latanoprost but by BAK, when comparing a BAK-preserved solution of latanoprost with a preservative-free latanoprost solution [37]. Therefore, preservatives are considered a major factor underlying the observed difference in cytotoxicity as Lat/T-FDC is preserved with a high concentration of BAK (0.02%). Taf/T-FDC includes lower concentrations of BAK (0.001%) than that of Lat/T-FDC, which reportedly do not affect cell viability [38], compared to Lat/T-FDC as demonstrated by the lower observed cytotoxicity of Taf/T-FDC compared to Lat/T-FDC. These findings indicate that Taf/T-FDC may be associated with lower ocular surface damage compared to Lat/T-FDC in clinical use, and superiority of Taf/T-FDC is thought to be associated in low BAK concentration of the formulation. It should be mentioned that this finding is based on *in vitro* assays. Further evaluations, including studies in animals, are needed to confirm its clinical relavence.

In conclusion, the results of both *in vivo* and *in vitro* studies demonstrate Taf/T-FDC as superior to Lat/T-FDC in terms of IOP-lowering effect duration and ocular surface toxicity. These results suggest that Taf/T-FDC is a promising FDC option in the treatment of glaucoma.

## Supporting Information

S1 File Monkey IOP data used in Fig 2. (XLSX)

S2 File Pharmacokinetics data used in Fig 3. (XLSX)

S3 File Pharmacokinetics data used in Fig 4. (XLSX)

S4 File Cell viability data used in Fig 5. (XLSX)
